# Suture residues are associated with histomorphological changes in failed anterior cruciate ligament hamstring autografts

**DOI:** 10.1002/jeo2.70811

**Published:** 2026-06-23

**Authors:** Steffen F. Siemoneit, Alexander Bosse, Steffen T. Ubl, Daniel Günther, Julius M. Wehrmann, Bertil Bouillon, Maiti Münchgesang, Thomas R. Pfeiffer

**Affiliations:** ^1^ Department of Orthopedic Surgery, Trauma Surgery and Sports Medicine, Cologne Merheim Medical Center Witten/Herdecke University Cologne Germany; ^2^ Department of Pathology, Klinikum Stuttgart University of Tübingen Tübingen Germany

**Keywords:** ACL revision histology, foreign body reactions, knee regression phenomena

## Abstract

**Purpose:**

This study aimed to evaluate histomorphological changes associated with residual foreign material in failed anterior cruciate ligament (ACL) autografts. We hypothesised that retained suture material is associated with histopathological regression phenomena in ligamentous and osseous compartments following ACL reconstruction failure.

**Methods:**

In this prospective study, 43 consecutive patients (mean age 29.7 ± 9.0 years; 20.9% female) undergoing revision surgery after failed autologous ACL reconstruction were included and analysed. A total of 76 bone‐graft specimens were obtained by over‐reaming femoral and tibial drill tunnels to retrieve the periarticular tunnel segment. Specimens were processed using Hematoxylin–Eosin, Masson–Goldner and Elastika–van Gieson staining. Morphological alterations related to foreign material were assessed qualitatively and semi‐quantitatively using a four‐point grading system under light microscopy. Evaluations were performed independently by two observers (a pathology professor and a trained investigator), with consensus in case of disagreement.

**Results:**

Foreign material residues were identified in 92.6% of specimens. Polarised light microscopy revealed birefringent suture remnants frequently associated with histiocytic inflammatory reactions and multinucleated giant cells. Observed regression phenomena included inflammatory reactions (IR), capillary proliferation (CP), osteonecrosis (ON), fissural splitting (FS) and intraligamentous cyst formation. Severe tissue destruction of the autograft or bony compartment was present in approximately 20% of femoral and tibial samples. Descriptive contingency analysis indicated that regression phenomena predominantly occurred in association with adjacent foreign body reactions, whereas isolated regression changes without detectable foreign material were rare.

**Conclusion:**

Residual suture material in failed ACL reconstructions is frequently associated with inflammatory and degenerative histomorphological changes in both tendon graft and bone tunnel compartments. Although causality cannot be established, these findings suggest that foreign body reactions may contribute to local tissue degradation. Further studies are needed to clarify the clinical and biomechanical relevance of these observations.

**Level of Evidence:**

N/A.

**Abbreviations**
ACL: anterior cruciate ligament
CP: capillary proliferation
EDTA: ethylenediaminetetraacetic acid
FBR: foreign body reaction
FFPE: formalin‐fixed paraffin‐embedded
FS: fissural splitting
HE: hematoxylin and eosin
IR: inflammatory reaction(s)
LARS: ligament augmentation and reconstruction system
MARS: multicenter ACL revision study
ON: osteonecrosis
PEEK: polyetheretherketone

## INTRODUCTION

Graft failure rates after anterior cruciate ligament (ACL) reconstruction have been reported to range from 2% to 10%, with higher figures reaching 25% in high‐risk populations. Notably, failure rates appear elevated in male patients relative to females, posing an ongoing challenge in clinical practice [8, 10, 11]. The MARS Group identified several potential causes of ACL revision: trauma (32%), technical errors—primarily mispositioned femoral tunnels (24%), biological failure (7%), combined factors (37%) and infection (<1%) [12]. The term ‘biological failure’ denotes graft failure occurring without identifiable surgical, biomechanical or traumatic aetiology. Unlike non‐anatomic tunnel placement, biological failure involves cellular‐ and histological‐level processes that are harder to detect. While autografts remain preferred for ACL reconstruction due to favourable surgical, clinical, functional and morphological outcomes, the aetiologies of graft failure are complex and not fully understood. Histopathological regression phenomena—collectively describing various microscopic structural alterations—may contribute to graft deterioration. A potential trigger for these morphological changes is the presence of foreign material within the graft environment, capable of eliciting localised tissue reactions. Such foreign body responses to suture materials have been documented in failed reconstructions across the Achilles and patellar tendons, hand flexor tendons, rotator cuff repairs and thyroid surgeries [2, 3, 4, 9, 12].

Thus, this study aims to evaluate the morphological changes associated with residual foreign material, particularly suture remnants, in failed ACL autografts. It was hypothesised that the presence of suture material may contributes to histopathological regression phenomena in the ligamentous and osseous compartments of the knee joint in cases of autologous graft failure.

## MATERIALS AND METHODS

### Study population and examination material

Baseline characteristics included age and gender. Inclusion criteria were as follows: (1) Primary ACL graft failure, regardless of its cause; (2) single ligament injury; (3) specimens obtained from femoral and tibial drill tunnels and from the intra‐articular ACL remnant; (4) sufficient quantity of tissue for staining; (5) Hamstring autografts. Samples with inadequate amounts of material or patients with chronic cardiovascular and renal diseases, metabolic disorders, thrombosis, allergies or tumours were excluded.

During arthroscopic ACL revision surgery, a total of 76 samples were harvested by drilling over the old tibial and femoral bony drill tunnels with a hollow reamer (diameter 10 mm × 2 mm) in the exact insertion area of the failed autograft to gain the periarticular (near‐joint) portion autograft covered by spongy or cortical bone.

**Figure 1:**
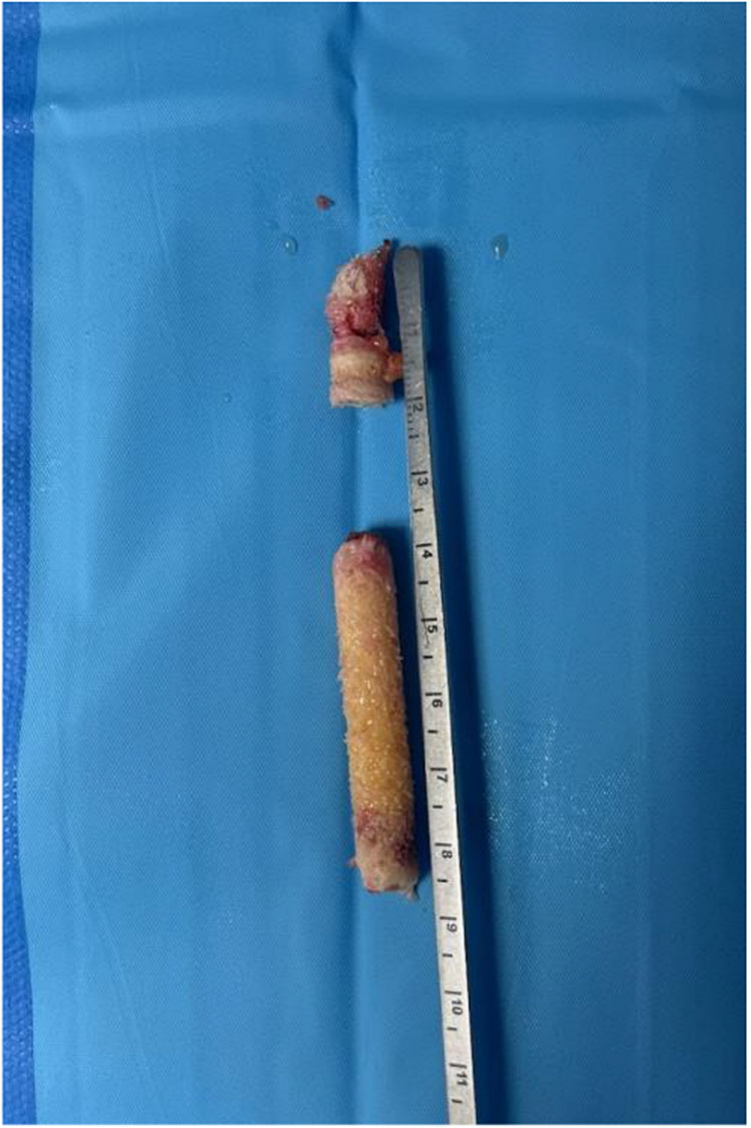
9‐mm diameter bone cylinder femoral (top) tibial (bottom).

The bone cylinder (Figure 1) is removed inside the hollow reamer. Subsequently, the drill channels were filled with bone material. The original specimens were fixed in 4% formalin solution and subsequently sent to the local Institute of Pathology for histological examination.

### Specimen preparation

**Figure 2:**
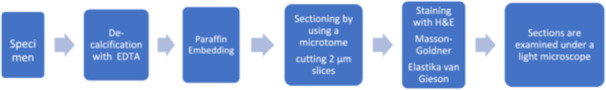
Specimen preparation. EDTA, ethylenediaminetetraacetic acid.

The 4% formalin‐fixed paraffin‐embedded (FFPE) specimens were prepared according to a defined protocol. Bone blocks were first decalcified with ethylenediaminetetraacetic acid (EDTA) and then embedded in paraffin. Subsequently, 2‐µm‐thick sections were cut with a microtome and stained microscopically with HE, Masson–Goldner and Elastika–Gieson (Figure 2). Finally, the preparations were analysed and evaluated by light microscopy (Zeiss, Germany) with 2.5×/5×/10×/20×/40× eyepieces at a tube factor of 1.25.

### Outcome measurements

Following light microscopic assessment, specimens were analysed for potential foreign body reactions (FBRs) as a stimulus for further histomorphological changes (Table 1) [11]. Specimens were categorised as femur, tibia and ACL remnant. Assessments were performed by a trained medical investigator with expertise in histomorphology and a professor of pathology. The severity of each observation was graded using a study‐specific grading method for each field of view. This grading method was qualitative and semi‐qualitative in nature; discrepancies were resolved by consensus.

**Table 1:** Description of histomorphological changes in autologous tendon grafts and bone tunnels.

Foreign material	Residual synthetic material visible under polarised light due to birefringence
Inflammation	Presence of histiocytic inflammatory cells infiltrating the graft or surrounding bone tunnel.
Capillary proliferation	Neoangiogenesis with dense capillary networks, often seen as part of chronic inflammatory response.
Osteonecrosis	Small, non‐vital bone trabeculae lacking osteocyte nuclei, indicating avascular bone necrosis.
Cysts	Large, round cystic spaces or tendinolyses within the autologous tendon graft.
Fissural splitting	Loosening and longitudinal splitting of collagen fibres within the tendon graft.

Statistical analysis was primarily descriptive and exploratory. Variables were summarised using contingency tables, reporting absolute and relative frequencies. For inferential analysis, findings were dichotomised into absence (0) versus presence (mild, moderate or severe).

Additionally, descriptive analyses were conducted to evaluate the frequency with which specific regression phenomena (FS, fissural splitting; ON, osteonecrosis; IR, inflammatory reactions; CP, capillary proliferation) occurred in association with FBR (yes/no). Associations between variables were examined using the two‐sided Fisher's exact test.

The significance level was set at 5% (*α* = 0.05). Given the exploratory nature of the study, inferential results should be interpreted as hypothesis‐generating rather than confirmatory. Statistical analyses were performed using IBM SPSS Statistics for Windows, Version 30.0 (IBM Corp.). An AI‐supported programme was employed to rectify grammatical errors.

## RESULTS

### Patient collection

The patient cohort comprised 43 individuals (9 female and 34 male) who underwent revision surgery of the ACL between 2017 and 2019. The age range was 18–58 years, with a mean of 29.67 years and a standard deviation of 9.02 years. The gender distribution included 9 women (20.9%) and 34 men (79.1%). No relevant comorbidities affecting tissue metabolism were documented. Seventeen of the patients had a history of smoking. All used autografts were hamstrings. The interval between primary and revision surgery ranged from 5.4 months to 18.1 years. Spontaneous graft failure occurred in one case (2.5%), whereas low‐energy trauma (e.g., running) accounted for 7.5% of cases. The majority of re‐ruptures (90%) were associated with adequate trauma during pivoting sports.

### Tissue sampling

The analysis yielded 30 samples resulting from the femoral drill tunnels, 39 from the tibial drill tunnels, and seven were intra‐articular ACL remnants. The variation in distribution can be attributed to the exclusion of material. This is particularly evident in the low number of ACL remnants.

### Histopathological findings

Histomorphological examination of ACL graft failure revealed a wide range of different regression phenomena directly associated with residual suture material. A total of five regression phenomena were identified, consisting of inflammatory reactions accompanied by capillary proliferation, osteonecrosis, intraligamentous cysts and fissural splitting.

**Figure 3:**
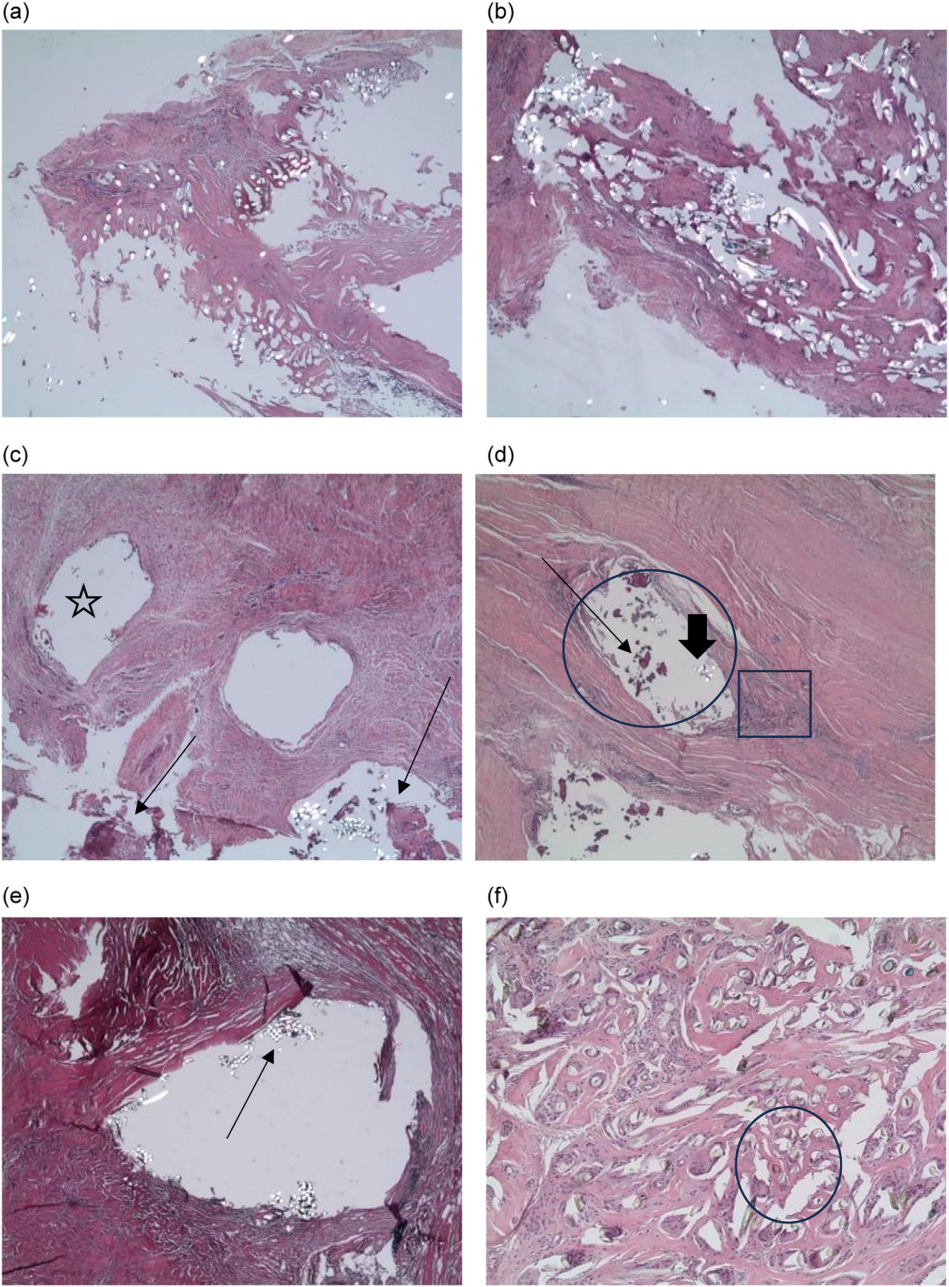
Foreign body reactions to suture residuals: (a) Clear accumulation of double light‐refracting suture remnants is evident, accompanied by a notable destruction of the tendon structure (HE ×2.5). (b) Suture material remnants causing severe tendinolysis and cystic alterations (HE ×5). (c) Large Cyst formation and tendinolysis with localised, partially resorbed foreign material (asterisk). Furthermore, necrotic changes of the bone (arrow) (HE ×5). (d) Intratendinous cysts (circle) with osteonecrosis (arrow), surrounding inflammatory reaction (box) and small remnants of birefringent foreign material (fat arrow) (HE ×10). (e) Cyst formation with tendinolysis and fissural splitting of tendon tissue due to embedded suture debris (arrow) (HE ×20). (f) The magnification shows a diffusely embedded foreign body with a high degree of destruction and evidence of numerous embedded multinucleated giant cells of foreign body type and partially incorporated suture material (circle) (HE ×20).

The utilisation of the polarisation technique facilitates the visualisation of embedded foreign material, which manifests as small nodular or diffuse formations (Figure 3a–f). These are often accompanied by inflammatory histiocytic reactions and necrotic alterations in the tendon and bone structures (Figure 3c,d). Reactive fissure splitting, cystic degeneration and tendinolysis are frequently observed (Figure 3b,e). Furthermore, giant cells that have phagocytosed foreign material are present with higher magnifications (Figure 3f).

### Qualitative assessment of FBRs

**Figure 4:**
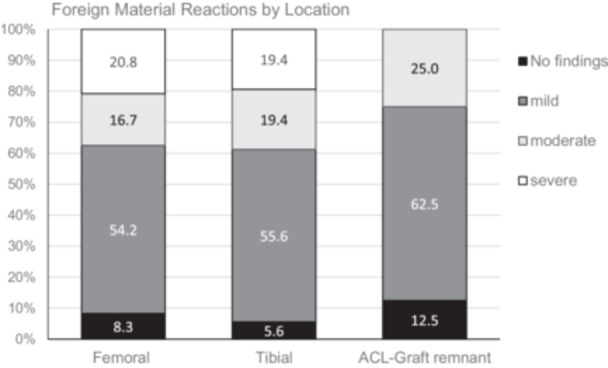
Distribution of foreign material reactions. ACL, anterior cruciate ligament.

The qualitative characteristics of the FBRs demonstrate that direct reactions to the underlying foreign material were observed in 92.6% of the specimens analysed. In 20.8% of cases, the most severe form of destruction was evident femorally, while in 19.4% of cases, it was evident in the tibia. The distribution of reaction severity is summarised in Table 2 and Figure 4.

**Table 2:** Qualitative distribution of foreign material reactions.

	Mild (%)	Moderate (%)	Severe (%)	No findings (%)
Femoral	54.2	16.7	20.8	8.3
tibial	55.6	19.4	19.4	5.6
ACL‐Graft remnant	62.5	25	0	12.5

Abbreviation: ACL, anterior cruciate ligament.

The descriptive analysis using contingency tables reveals an association between manifestations of foreign material reactions and regression phenomena in the tibial and femoral compartments.

**Figure 5:**
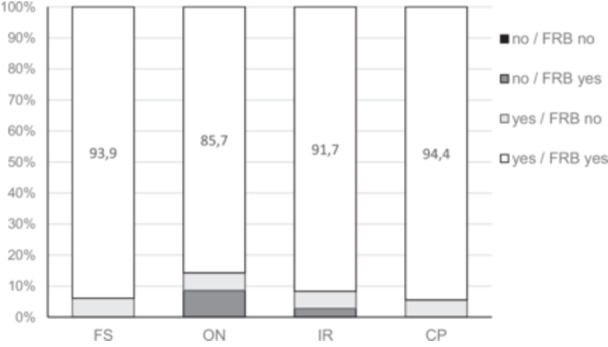
Tibial distribution of regression phenomena in relation to FBRs. CP, capillary proliferations; FRB, foreign body reaction; FS, fissural splitting; IR, inflammatory reactions; ON, osteonecrosis.

**Figure 6:**
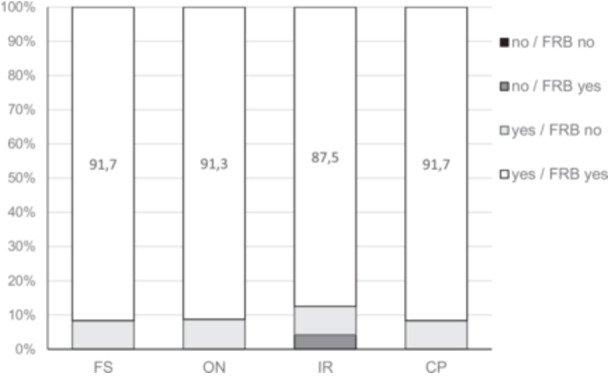
Femoral distribution of regression phenomena in relation to FBRs. CP, capillary proliferations; FRB, foreign body reaction; FS, fissural splitting; IR, inflammatory reactions; ON, osteonecrosis.

It is evident that the most prevalent occurrence is the combination of local FBRs with accompanying regression phenomena (a minimum of 85.7%), which is distributed uniformly across the various regression types. Conversely, the occurrence of regression phenomena without adjacent FBRs is observed in less than 10% of all cases (Figures 5 and 6). Due to the limited number of cases, no statistically significant associations were detected.

## DISCUSSION

### Reaction to suture remnants

The most important finding of the present study is the frequent close association between residual suture material and inflammatory‐degenerative regression phenomena in failed human ACL autografts. Foreign‐body reactions associated with residual foreign material were identified in 92.6% of all analysed specimens, supporting the hypothesis that these reactions may represent a previously underrecognized local factor associated with graft degeneration and impaired graft‐bone integration. Polarised light microscopy demonstrated birefringent foreign material morphologically consistent with residual suture material rather than abraded fixation‐device particles. The identified remnants were predominantly compatible with resorbable or semi‐resorbable polyfilament sutures.

The observed regression phenomena within both ligamentous and osseous compartments frequently occurred adjacent to residual foreign material and may reflect chronic local tissue reactions. Severe tendinolysis and cystic degeneration associated with embedded suture remnants were frequently observed (Figure 3a,b,e), while osteonecrotic alterations and surrounding inflammatory reactions were detectable in the adjacent osseous compartment (Figure 3c,d). Histological findings including histiocytic inflammation and multinucleated giant cells were consistent with previously described foreign‐body responses to suture material in other orthopaedic and soft‐tissue procedures. At higher magnification, macrophages containing phagocytosed foreign material were detectable, supporting the presence of an ongoing local inflammatory response (Figure 3f).

**Figure 7:**
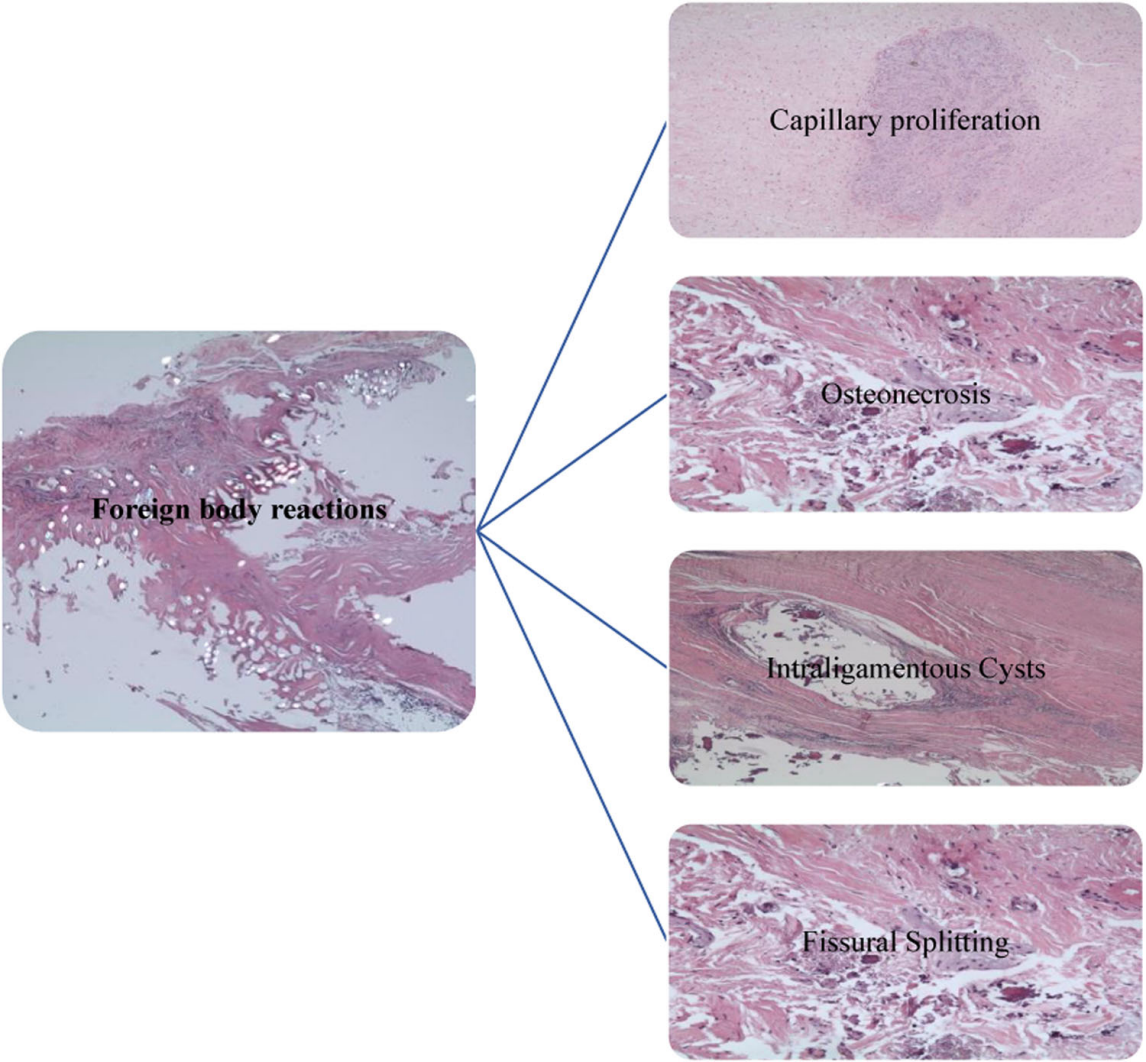
Residual foreign material as a potential pathological factor for regressive changes in anterior cruciate ligament‐graft failure.

Descriptive contingency analysis further demonstrated that regression phenomena predominantly occurred in close association with foreign‐body reactions, whereas isolated regression changes without detectable foreign material were comparatively uncommon (Figures 5 and 6). However, due to the observational design and limited sample size, no causal relationship can be established. As illustrated in Figure 7, residual suture material may therefore represent a potential contributing factor to the observed histomorphological alterations in failed ACL grafts.

Future studies should focus on elucidating the extent to which these histopathological changes impact the mechanical stability and long‐term success of ACL reconstructions.

Similar reactions to residual suture material have been observed in failed reconstructions of the Achilles tendon [10]. Four years post‐surgery, the Ethibond‐repaired patellar tendon failed, with histology revealing a foreign body giant cell reaction around the suture and necrotic tendon with polymorphonuclear infiltration [3]. In hand surgery, several cases have been reported in which use of the FiberLoop device for tendon repair resulted in granuloma formation and a pronounced foreign body inflammatory response [2]. Furthermore, histological analysis of failed Suretac devices in the context of rotator cuff reconstruction also revealed histiocytic inflammatory infiltrates with giant cells and birefringent foreign material [4]. The analysis of silk sutures in the area of the thyroid gland demonstrated a comparable pattern with severe inflammation [9]. Especially, the use of polyethylene sutures has been observed to result in pronounced granulomatous changes with accumulated giant cells, which are also interpreted as a clear risk of graft failure. Moreover, inflammatory response is more pronounced with polyfilament sutures than with monofilament sutures [7]. Interestingly, the described multinucleated giant cells have the capacity to differentiate into functional osteoclasts when stimulated by surgical suture material—a process which can result in osteolysis and implant loosening [6]. A review of the literature on fascial closure following laparoscopic cholecystectomy reveals that histological reactions to fascial sutures, including pus and fibrosis, are observed approximately 90 days post‐surgery [5].

### Reactions to fixation material or synthetic transplants

This study focused on suture material residues, yet the literature shows that foreign‐body reactions also occur with other fixation materials. Interference screws can provoke regression phenomena with similar histological patterns, including macrophage proliferation, multinucleated giant cells, and occasional degenerative or cystic changes [8, 10, 13]. Complete remodelling of biodegradable screws into bone has not been reported during follow‐up [14]. A case report further described marked osteolysis after PEEK screw implantation, with a histiocytic inflammatory response comparable to that seen with suture residues [5].

Comparable findings appear in analyses of synthetic graft failure [14]. Implantation of synthetic ligaments (LARS) has been associated with chronic synovial inflammation, fibrosis and giant cells containing phagocytosed material, indicating a foreign‐body reaction, as well as poor graft–bone integration [1, 8]. It can be hypothesised that not only suture material residues but also fixation materials have the potential to induce histomorphological regression.

### Limitations

This study has several limitations. First, the absence of a control group of intact ACL grafts limits the ability to distinguish pathological from physiological remodelling processes. Second, a study‐specific semi‐quantitative grading system was used, which may affect reproducibility. Third, the relatively small sample size may limit the detection of less frequent changes. Fourth, variability in the time interval between graft failure and revision surgery may have influenced the observed morphology. Finally, the observational design precludes conclusions regarding causality.

## CONCLUSION

Residual suture material in failed ACL reconstructions is frequently associated with inflammatory and degenerative histomorphological changes, including osteonecrosis, cyst formation and structural graft alterations. These findings suggest that FBRs may represent a contributing local factor in graft degeneration. However, their impact on mechanical stability and clinical failure remains unclear.

Reducing unnecessary suture material and avoiding its placement at the graft–bone interface may be considered as potential strategies to minimise adverse tissue reactions. Further studies are required to clarify the clinical and biomechanical relevance of these findings.

## AUTHOR CONTRIBUTIONS

Steffen F. Siemoneit, Thomas R. Pfeiffer and Alexander Bosse were involved in the design of the study, data collection and carrying out the histomorphological investigations as well as quantitative measurements. The complete article was written by Steffen F. Siemoneit. Daniel Günther was particularly involved in the design and structure of the article. Steffen T. Ubl, Thomas R. Pfeiffer, Daniel Günther, Julius M. Wehrmann, Bertil Bouillon, Maiti Münchgesang and Alexander Bosse critically revised the article. Previous versions of the manuscript were commented by all authors. All authors have read and approved the manuscript.

## FUNDING INFORMATION

The authors have no funding to report.

## CONFLICT OF INTEREST STATEMENT

The authors declare no conflicts of interest.

## ETHICS STATEMENT

The study was approved by the Institutional Review Board and ethics committee of the University of Tuebingen (ethics proposal number 684/2019BO2).

## Data Availability

The datasets generated and analysed during the current study are available from the corresponding author on request. However, to protect patient privacy and comply with ethical regulations, any personally identifiable information has been excluded and will not be shared.

## References

[1] Ambrosio L , Vadalà G , Castaldo R , Gentile G , Nibid L , Rabitti C , et al. Massive foreign body reaction and osteolysis following primary anterior cruciate ligament reconstruction with the ligament augmentation and reconstruction system (LARS): a case report with histopathological and physicochemical analysis. BMC Musculoskelet Disord. 2022;23(1):1140. 36581922 10.1186/s12891-022-05984-5 PMC9801556

[2] Arner M , Unge L , Franko MA , Svingen J . Inflammatory reaction to suture materials after flexor tendon repair. A retrospective study of 594 patients. Case Rep Plastic Surg Hand Surg. 2023;10(1):2222807. 10.1080/23320885.2023.2222807 PMC10283439 37351525

[3] Blasco A , Baixauli E . Granuloma formation associated with patellar tendon necrosis in response to Ethibond confirmed by histopathological examination. BMJ Case Rep. 2018;2018:bcr‐2017‐222854. 10.1136/bcr-2017-222854 PMC5950563 29754129

[4] Burkart A , Imhoff AB , Roscher E . Foreign‐body reaction to the bioabsorbable suretac device. Arthrosc J Arthrosc Rel Surg. 2000;16(1):91–95. 10.1016/s0749-8063(00)90134-8 10627352

[5] Butler JJ , Shukhmakher E , Hartman H , Kennedy JG . Talar and fibular histiocytic‐driven massive expansile osteolysis following polyetheretherketone interference screw implantation: a case report. J ISAKOS. 2024;9(3):410–414. 38266965 10.1016/j.jisako.2024.01.009

[6] Dapunt U , Prior B , Kretzer JP , Hänsch GM , Gaida MM . The effect of surgical suture material on osteoclast generation and implant‐loosening. Int J Med Sci. 2021;18(2):295–303. 33390798 10.7150/ijms.50270 PMC7757137

[7] Dragovic M , Pejovic M , Stepic J , Colic S , Dozic B , Dragovic S , et al. Comparison of four different suture materials in respect to oral wound healing, microbial colonization, tissue reaction and clinical features—randomized clinical study. Clin Oral Investig. 2020;24(4):1527–1541. 10.1007/s00784-019-03034-4 31342245

[8] Grassi A , Kim C , Marcheggiani Muccioli GM , Zaffagnini S , Amendola A . What is the mid‐term failure rate of revision ACL reconstruction? A systematic review. Clin Orthop Relat Res. 2017;475(10):2484–2499. 28493217 10.1007/s11999-017-5379-5 PMC5599393

[9] Hocwald E , Sichel JY , Dano I , Meir K , Eliashar R . Adverse reaction to surgical sutures in thyroid surgery. Head Neck. 2003;25(1):77–81. 12478548 10.1002/hed.10157

[10] Ollivere BJ , Bosman HA , Bearcroft PWP , Robinson AHN . Foreign body granulomatous reaction associated with polyethelene ‘Fiberwire®’ suture material used in Achilles tendon repair. Foot Ankle Surg. 2014;20(2):e27–e29. 24796842 10.1016/j.fas.2014.01.006

[11] Siemoneit SF , Bosse A , Ubl ST , Günther D , Wehrmann JM , Bouillon B , et al. Histomorphological changes in autologous anterior cruciate ligament graft failure show predominantly inflammatory reactions, fissure defects and osteonecrosis. J Exp Orthop. 2025;12(4):e70545. 41323544 10.1002/jeo2.70545 PMC12661208

[12] The MARS Group , Wright RW , Huston LJ , Spindler KP , Dunn WR , Haas AK , Allen CR , et al. Descriptive epidemiology of the multicenter ACL revision study (MARS) cohort. Am J Sports Med. 2010;38(10):1979–1986. 20889962 10.1177/0363546510378645 PMC3655411

[13] Umar M , Baqai N , Peck C . Foreign body reaction to a bioabsorbable interference screw after anterior cruciate ligament reconstruction. BMJ Case Rep. 2009;2009:bcr0920081007. 10.1136/bcr.09.2008.1007 PMC3027541 21686509

[14] Weiler A , Hoffmann RF , Stähelin AC , Helling H‐J , Südkamp NP . Biodegradable implants in sports medicine: the biological base. Arthrosc J Arthrosc Rel Surg. 2000;16(3):305–321. 10.1016/s0749-8063(00)90055-0 10750011

